# The predictive value of the hs-CRP/HDL-C ratio, an inflammation-lipid composite marker, for cardiovascular disease in middle-aged and elderly people: evidence from a large national cohort study

**DOI:** 10.1186/s12944-024-02055-7

**Published:** 2024-03-01

**Authors:** Yu Gao, Miyuan Wang, Ruiting Wang, Jinchi Jiang, Yueyao Hu, Wei Wang, Yong Wang, Haijing Li

**Affiliations:** 1 College of Chinese Medicine, Beijing University of Chinese Medicine, Beijing, 100029 China; 2 Key Laboratory of TCM Syndrome and Formula, Beijing University of Chinese Medicine, Ministry of Education, Beijing, 100029 China; 3 Department of Traditional Chinese Medicine, Peking University Third Hospital, Beijing, 100191 China; 4 School of public health, Huazhong University of Science and Technology, Hubei, 430074 China; 5 Dongzhimen Hospital, Beijing University of Chinese Medicine, Beijing, 100029 China; 6 Guangzhou University of Chinese Medicine, Guangdong, 510006 China; 7 School of Life Sciences, Beijing University of Chinese Medicine, Beijing, 100029 China

**Keywords:** C-reactive protein, High-density lipoprotein cholesterol, Cardiovascular disease, Cohort study, Risk factor

## Abstract

**Background and aims:**

Cardiovascular disease (CVD) is associated with inflammation and abnormal lipid metabolism. However, a single inflammatory index or a single lipid index cannot accurately predict the prognosis of CVD independently because it is prone to be affected by various confounding factors.

**Methods:**

This population-based cohort study included 6,554 participants from the China Health and Retirement Longitudinal Study (CHARLS) to investigate correlations. In the present study, the occurrence of CVD events such as stroke and heart disease was evaluated by considering self-reported diagnoses at the beginning of the study and during wave 4, and a restricted cubic spline model was used to investigate potential nonlinear relationships in addition to multivariate logistic regression models. Stratified analyses were performed to examine how sociodemographic characteristics may influence the results.

**Results:**

Seven years of follow-up (2011–2018) revealed that 786 people (11.99%) developed CVD. According to the adjusted model, the high-sensitivity C-reactive protein (hs-CRP)-to-high-density lipoprotein cholesterol (HDL-C) ratio is a contributing factor to CVD risk (OR 1.31, 95% CI 1.05–1.64). In addition, a nonlinear relationship was observed between the hs-CRP/HDL-C ratio and the occurrence of new CVD, stroke, or cardiac issues (*P*_overall_ <0.05, _Pnonlinear_ <0.05). Moreover, noteworthy associations between the hs-CRP/HDL-C ratio and age were detected in the stratified analysis (*P* = 0.048), indicating that younger participants had more negative effects of a high hs-CRP/HDL-C ratio.

**Conclusions:**

According to the present cohort study, a high hs-CRP/HDL-C ratio is a significant risk factor for CVD, new stroke, and heart problems. Early intervention in patients with increased hs-CRP/HDL-C ratios may further reduce the incidence of CVD, in addition to focusing on independent lipid markers or independent inflammatory markers.

**Supplementary Information:**

The online version contains supplementary material available at 10.1186/s12944-024-02055-7.

## Introduction

Cardiovascular diseases (CVDs) encompass a range of medical conditions primarily including myocardial ischaemia, heart attack, angina pectoris, impaired cardiac function, irregular heartbeat, stroke and associated ailments [1]. A 2021 study on cardiovascular well-being and illness in China revealed that CVDs were responsible for approximately 46.74% of the total mortality rate in rural areas but accounted for approximately 44.26% of the total mortality rate in urban areas in 2019. This finding implies that CVD contributes to two-fifths of all deaths in China. Approximately 330 million people suffer from CVD worldwide, with a specific focus on the prevalence of coronary heart disease, which affects approximately 11.39 million individuals [2]. Approximately 30% of the yearly deaths worldwide (equivalent to 17.6 million individuals annually) and approximately 10% of the global disease burden can be attributed to CVDs [3, 4]. Despite advancements in medical technology and the maturity of interventional technology, millions of people worldwide still die from acute myocardial infarction (AMI) each year [5, 6]. Patients with severe AMI also have higher mortality and more comorbidities, particularly adults in their middle and later years.

The occurrence of CVD depends on age, sex, genetic predisposition, lifestyle and other factors. Blood lipids, such as low-density lipoprotein cholesterol (LDL-C) and triglycerides (TGs), can predict CVD risk and prognosis. Research has indicated a direct relationship between high-density lipoprotein cholesterol (HDL-C) and the presence of coronary artery stenosis [7]. HDL-C can prevent cholesterol transport, protect vascular endothelial cells, and have antioxidant effects [7–9]. The HDL-C level exhibits an inverse relationship with the risk of CVD [10]. However, clinical trials to improve the prognosis of patients with CVD by increasing the level of HDL-C have failed, which has raised doubts about the protective effect of HDL-C [11]. Another key element in the development of atherosclerosis is inflammation. In addition, when exposed to various risk factors, inflammation can cause plaque rupture, leading to myocardial infarction characterized by ST-segment elevation [12]. Furthermore, increased mortality following myocardial infarction has been associated with the presence of inflammation [13]. Numerous inflammatory markers, including the platelet count, neutrophil count, C-reactive protein (CRP) level, and high-sensitivity C-reactive protein (hs-CRP) level, have demonstrated potential for short- or long-term CVD prediction in patients with cardiovascular conditions [14–18]. Numerous clinical investigations of compliance markers have been conducted to further increase the predictive accuracy of these markers. The neutrophil-to-lymphocyte ratio was shown to be an autonomous determinant of coronary artery disease (CAD) by Zsolt Bagyura et al. [19]. According to Mehmet Serkan Cetin et al., the ratio of monocytes to HDL-C in patients with AMI could serve as an indicator of the extent of CAD and the potential occurrence of significant cardiovascular complications [20]. The ratio of TGs to HDL-C is linked to increased CVD risk according to Bizhong Che et al. [21].

Numerous research endeavours have examined risk prediction models for CVD, with a particular emphasis on independent inflammatory or cholesterol indices. Studies have indicated a link between decreased concentrations of HDL-C and hs-CRP and the emergence and progression of CVD. These two markers are inexpensive and simple to use in clinical practice. The use of inflammation-lipid metrics may improve the prognostic prediction of CVD due to the association between inflammation and lipid metabolism [20]. Thus, the objective of this comprehensive study was to evaluate the capacity of the hs-CRP/HDL-C index to predict CVD occurrence among individuals in the middle and later stages of life residing within a community setting in China.

## Methods

### Study design and population

The study utilized data from the China Health and Retirement Longitudinal Study (CHARLS), an ongoing research project conducted by the National Development Research Institute (China Center for Economic Research). The primary objective of the CHARLS is to promote interdisciplinary ageing research and investigate demographic changes in China’s population [22]. A collection of excellent microdata reflecting Chinese households and individuals aged 45 years and older was gathered by the CHARLS. One year after the conclusion of data collection, all data are made publicly available and thereafter available to the academic community. The collection of CHARLS data was ethically approved by the Peking University Biomedical Ethics Review Board (IRB00001052-11015), and the study protocol complied with the 1975 Declaration of Helsinki’s ethical standards. All participants in the research provided their consent after being given detailed written information.

**Fig. 1:**
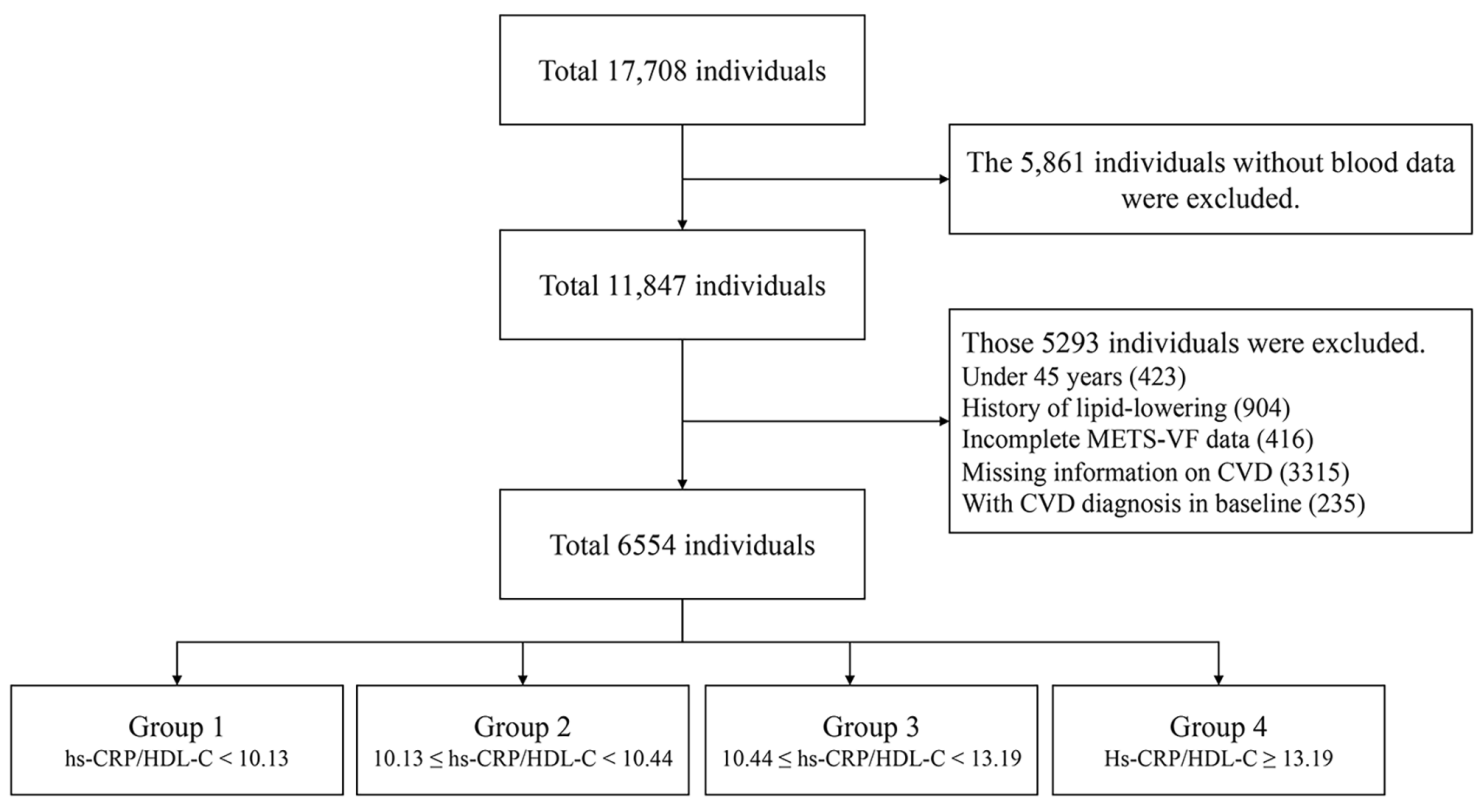
The flow chart illustrates the participant selection process in the CHARLS cohort

The procedures followed in this investigation were in conformity with the relevant CHARLS requirements and recommendations. This research studied patient blood samples and associated sociodemographic characteristics collected from Wave 1 (2011) to Wave 4 (2018) of the CHARLS over a total follow-up period of 7 years. Of the participants surveyed at baseline in 2011, 17,708 completed the baseline physical examination and questionnaires, and blood data were available for 11,847 participants. This study included this sample of participants to examine the association between CVD incidence and the hs-CRP/HDL-C ratio. An age under 45 years, a history of lipid-lowering and hypoglycaemic drug use, insufficient METS-VF data, missing information on cardiovascular events in waves 2011 and 2018, and the presence of cardiovascular events at baseline were among the factors used to exclude participants. After screening based on the above criteria, a total of 6,554 eligible subjects were included at baseline and assessed for incident CVD, the hs-CRP/HDL-C ratio and other covariates (Fig. 1).

### Assessment of the hs-CRP/HDL-C ratio

Hs-CRP and HDL-C levels were measured in blood samples stored in a refrigerator using standard enzyme colorimetric assays at the Youanmen Center for Clinical Laboratory of Capital Medical University (see Supplementary material 1). In this investigation, the hs-CRP/HDL-C ratio was determined by dividing the hs-CRP level (mg/L) by the HDL-C level (mg/dL/1000). Based on the quartiles for the hs-CRP to HDL-C ratio, the participants were split into four categories: Group 1, an hs-CRP/HDL-C ratio < 10.13; Group 2, 10.13 ≤ hs-CRP/HDL-C ratio < 10.44; Group 3, 10.44 ≤ hs-CRP/HDL-C ratio < 13.19; and Group 4, an hs-CRP/HDL-C ratio ≥ 13.19.

### Assessment of Incident CVD

The study’s definition of CVD included heart disease, coronary artery disease, angina pectoris, heart failure or other cardiac problems, and stroke, as well as a self-reported medical diagnosis of CVD. Three follow-up surveys (Waves 2, 3, and 4) confirmed the occurrence of CVD events. Participants were questioned twice about CVD events, at baseline and during follow-up at WAVE 4, as follows: “Have you ever had a heart condition such as coronary artery disease, angina pectoris, congestive heart failure or other heart issues diagnosed by a doctor?” or “Have you ever had a stroke diagnosed by a doctor?” CVD events were recorded for participants who self-reported having experienced at least one heart attack or stroke episode during the CHARLS follow-up period (0 = no, 1 = yes). Secondary outcomes, including heart disease and stroke, were independent components of the primary outcome. All the conditions were assessed independently by trained investigators.

### Assessment of covariates

Based on previous research, this study also considered baseline demographic information and health-related behaviours, adjusting for these potentially confounding factors in the analyses [23]. The sociodemographic factors included age, sex (male or female), marital status (married, widowed, separated, divorced, or never married), education level (elementary school or less, high school, college or more) and location of residence (city/town or village). The definitions of city/town and village were based on the definitions of the National Statistical Office. Cities/towns were defined as areas located in cities or suburbs, towns or suburbs, or other special areas where nonagricultural industries account for more than 70% of the total; these areas include special economic zones and state-owned agribusinesses [22]. Physical and chemical markers (blood pressure, uric acid, LDL-C, and blood glucose levels) could be independent risk factors. Two additional factors considered in the analysis were the frequency of alcohol use (never, less than once a month, and more than once a month) and smoking status (never, past, and present smoking). Insufficient sleep was linked to a greater likelihood of developing CVD, as the sleep duration in individuals with metabolic syndrome was also taken into account (the criteria for metabolic syndrome according to the NCEP ATP III guidelines encompass central obesity, dyslipidaemia with atherogenic properties, hypertension, and hyperglycaemia/insulin resistance) [24, 25].

### Statistical analyses

The median and quartiles (Q1–Q3) are reported for nonnormally distributed data, while categorical variables are represented as proportions. Groups were created based on the hs-CRP/HDL-C quartiles according to the baseline characteristics. To perform the statistical comparisons, suitable tests such as chi-square tests, K-W tests, and ANOVA were employed as needed. The odds ratio (OR) and 95% confidence interval (CI) were calculated by three logistic regression models using continuous variables (per interquartile range (IQR) increase) or categorical variables (quartiles). To investigate potential nonlinear correlations, restrictive cubic spline analyses were conducted at the 5th, 35th, 65th, and 95th percentiles of the hs-CRP/HDL-C ratio. Additionally, the associations between the hs-CRP/HDL-C ratio and the occurrence of cardiovascular disease, stroke, and heart problems were graphically represented. Model 1 assessed the fundamental link between the hs-CRP/HDL-C ratio and the development of CVD; Model 2 further adjusted for sex, education level, residence location, and marital status; Model 3 further considered physical and chemical indicators (uric acid, LDL-C, blood glucose and blood pressure levels) as well as health behaviours (smoking status, alcohol intake, and duration of sleep). To evaluate how sociodemographic attributes and health-related activities affect the association between the hs-CRP/HDL-C ratio and the occurrence of CVD, separate interaction analyses were also conducted. To assess the impact of the multiplicative interaction factors incorporated into these models, alternative tests were performed using the likelihood ratio. To determine whether the hs-CRP/HDL-C ratio provides any supplementary predictive value beyond clinical risk variables already established based on C statistics, continuous net reclassification improvement (NRI), or integrated discrimination improvement (IDI), this study fitted the hs-CRP/HDL-C ratio to a logistic regression model.

R 4.1 was used for the statistical analyses. Utilizing the ‘ANOVA’ function from the rms R package, limited cubic splines were analysed. The level of statistical significance was determined using a two-tailed *P* value threshold of 0.05.

## Results

### Characteristics of the study population based on hs-CRP/HDL-C ratio quartiles

A total of 6,554 people (3,083 males and 3,471 females) with a median age of 57 years were included in this study. During the 7-year follow-up period (2011–2018), 786 individuals (11.99%) developed CVD. Based on the quartile groups of the hs-CRP/HDL-C ratio, the initial attributes of the study subjects are presented in Table 1. The results revealed a gradual increase in the incidence of new CVD among participants as their hs-CRP/HDL-C ratio increased (10.13 vs. 10.44 vs. 13.19 vs. 14.22, *P* < 0.01), with similar trends observed for both heart problems (6.71 vs. 6.59 vs. 9.10 vs. 8.72, *P* < 0.01) and stroke (3.90 vs. 4.4 vs. 4.95 vs. 6.53, *P* < 0.01). In addition, the results revealed statistically significant differences in age, sex, residence location, marital status, drinking status, smoking status, triglyceride levels, and HDL-C levels among the study participants (all *P* values < 0.05).

**Table 1:** Baseline characteristics of the study population according to the hs-CRP/HDL-C ratio

Variables	Total (*n* = 6554)	Q1 (*n* = 1639)	Q2 (*n* = 1638)	Q3 (*n* = 1638)	Q4 (*n* = 1639)	*P*
**Age, years, mean (Q1, Q3)**	57 (51, 63)	56 (49, 62)	57 (50, 63)	57 (51, 64)	58 (52, 65)	< 0.01
**Sex, n (%)**						< 0.01
Female	3471 (52.96)	928 (56.62)	869 (53.05)	831 (50.73)	843 (51.43)	
Male	3083 (47.04)	711 (43.38)	769 (46.95)	807 (49.27)	796 (48.57)	
**Marital status, n (%)**						0.01
Married	5911 (90.19)	1490 (90.91)	1501 (91.64)	1471 (89.8)	1449 (88.41)	
Non-Married	643 (9.81)	149 (9.09)	137 (8.36)	167 (10.2)	190 (11.59)	
**Education level, n (%)**						0.91
Elementary school and below	4514 (68.87)	1142 (69.68)	1124 (68.62)	1112 (67.89)	1136 (69.31)	
High school	1365 (20.83)	337 (20.56)	348 (21.25)	347 (21.18)	333 (20.32)	
College and higher	675 (10.3)	160 (9.76)	166 (10.13)	179 (10.93)	170 (10.37)	
**Location of residence, n (%)**						< 0.01
Urban	6135 (93.61)	1571 (95.85)	1522 (92.92)	1529 (93.35)	1513 (92.31)	
Rural	419 (6.39)	68 (4.15)	116 (7.08)	109 (6.65)	126 (7.69)	
**Smoking status, n (%)**						< 0.01
Never	4051 (61.81)	1058 (64.55)	1037 (63.31)	988 (60.32)	968 (59.06)	
Former smoker	461 (7.03)	90 (5.49)	106 (6.47)	122 (7.45)	143 (8.72)	
Current smoker	2042 (31.16)	491 (29.96)	495 (30.22)	528 (32.23)	528 (32.21)	
**Drinking status, n (%)**						0.02
None of these	4277 (65.26)	1042 (63.58)	1030 (62.88)	1087 (66.36)	1118 (68.21)	
Drinks, but less than once a month	553 (8.44)	142 (8.66)	138 (8.42)	145 (8.85)	128 (7.81)	
Drinks more than once a month	1724 (26.3)	455 (27.76)	470 (28.69)	406 (24.79)	393 (23.98)	
**Sleep duration, hours, mean (Q1, Q3)**	7 (5, 8)	7 (5, 8)	6.75 (5, 8)	7 (5, 8)	7 (5, 8)	0.86
**Developed heart problems, n (%)**						**< 0.01**
No	6044 (92.22)	1529 (93.29)	1530 (93.41)	1489 (90.9)	1496 (91.28)	
Yes	510 (7.78)	110 (6.71)	108 (6.59)	149 (9.1)	143 (8.72)	
**Developed stroke, n (%)**						< 0.01
No	6230 (95.06)	1575 (96.1)	1566 (95.6)	1557 (95.05)	1532 (93.47)	
Yes	324 (4.94)	64 (3.9)	72 (4.4)	81 (4.95)	107 (6.53)	
**Developed CVD, n (%)**						< 0.01
No	5768 (88.01)	1473 (89.87)	1467 (89.56)	1422 (86.81)	1406 (85.78)	
Yes	786 (11.99)	166 (10.13)	171 (10.44)	216 (13.19)	233 (14.22)	
**SBP (mmHg), mean (Q1, Q3)**	126 (114, 140)	122 (112, 137)	124 (113, 138)	127 (115, 141)	129 (116, 145)	< 0.01
**DBP (mmHg), mean (Q1, Q3)**	74 (67, 83)	73 (66, 81)	74 (67, 82)	75 (68, 83)	76 (69, 84)	< 0.01
**TG (mg/dl), mean (Q1, Q3)**	100 (72.57, 141.6)	83.19 (63.72, 113.28)	99.12 (72.57, 135.4)	109.74 (78.76, 153.99)	115.05 (80.54, 164.61)	< 0.01
**Uric acid (mg/dl), mean (Q1, Q3)**	4.24 (3.54, 5.08)	3.92 (3.32, 4.67)	4.10 (3.46, 5.00)	4.38 (3.68, 5.20)	4.56 (3.78, 5.40)	< 0.01
**LDL-C (mg/dl), mean (Q1, Q3)**	114.43 (94.72, 135.70)	111.73 (92.78, 132.41)	113.66 (95.10, 137.15)	118.30 (97.52, 139.18)	113.66 (92.40, 134.92)	< 0.01
**Glucose (mg/dl), mean (Q1, Q3)**	101.34 (93.96, 110.52)	99.72 (92.7, 107.64)	101.34 (94.14, 110.16)	101.7 (94.14, 110.52)	102.78 (95.22, 114.03)	< 0.01
**HDL-C (mg/dl), mean (Q1, Q3)**	50.65 (41.75, 60.7)	59.54 (51.03, 69.39)	51.42 (44.07, 60.7)	47.94 (40.21, 56.83)	44.07 (36.34, 52.58)	< 0.01
**hs-CRP (mg/l), mean (Q1, Q3)**	0.94 (0.51, 1.92)	0.37 (0.29, 0.46)	0.69 (0.56, 0.84)	1.28 (1.05, 1.60)	3.45 (2.41, 6.03)	< 0.01
**hs-CRP/HDL-C, mean (Q1, Q3)**	0.02 (0.01, 0.04)	0.01 (0.00, 0.01)	0.01 (0.01, 0.02)	0.03 (0.02, 0.03)	0.08 (0.05, 0.13)	< 0.01

CVD, cardiovascular disease; HDL-C, high-density lipoprotein cholesterol; hs-CRP, high-sensitivity C-reactive protein; LDL-C, low-density lipoprotein cholesterol; Q1, the first quartile of the CRP/HDL-C ratio; Q2, the median of the CRP/HDL-C ratio; Q3, the third quartile of the CRP/HDL-C ratio; Q4, the fourth quartile of the CRP/HDL-C ratio; TG, triglyceride

### Correlations between the initial hs-CRP/HDL-C ratio and the occurrence of CVD/stroke/cardiac issues

The link between the hs-CRP/HDL-C ratio and the likelihood of developing CVD/stroke/heart problems can be observed in Table 2. Model 3 was adjusted for all possible confounding variables (sex, education level, residence location, marital status, health behaviours, and physical and chemical indicators). The study findings indicated a direct association between the quartiles of the hs-CRP/HDL-C ratio and the occurrence of new cases of CVD (*P* for trend = 0.003). In comparison to participants with an hs-CRP/HDL-C ratio in the bottom 25% (Q1), those with an hs-CRP/HDL-C ratio in the top 25% (Q4) exhibited the greatest susceptibility to new-onset CVD (OR = 1.31, 95% CI 1.05–1.64). When the hs-CRP/HDL-C ratio was analysed as a continuous variable, the likelihood of developing new CVD was found to increase by 1.3% for each IQR increase in the hs-CRP/HDL-C ratio, but the difference was not statistically significant (OR = 1.013, 95% CI 0.994–1.033).

**Table 2:** Prospective associations between the baseline hs-CRP/HDL-C ratio and follow-up incident CVD/stroke/heart problems

	Model 1 ^a^	*P*	Model 2 ^b^	*P*	Model 3 ^c^	*P*
**CVD**						
hs-CRP/HDL-C ratio per IQR	1.019(1.001,1.038)	0.041	1.016(0.997,1.035)	0.090	1.013(0.994,1.033)	0.172
Q1	ref		ref		ref	
Q2	1.03 [0.83, 1.30]	0.769	1.02 [0.81, 1.27]	0.897	0.99 [0.79, 1.25]	0.962
Q3	1.35 [1.09, 1.67]	0.007	1.30 [1.05, 1.62]	0.018	1.24 [1.00, 1.55]	0.054
Q4	1.47 [1.19, 1.82]	< 0.001	1.40 [1.13, 1.73]	0.002	1.31 [1.05, 1.64]	0.016
*P* for trend	< 0.001		< 0.001		0.003	
**Stroke**						
hs-CRP/HDL-C ratio per IQR	1.029(1.005,1.053)	0.019	1.023(0.999,1.049)	0.065	1.018(0.992,1.044)	0.175
Q1	ref		ref		ref	
Q2	1.13 [0.80, 1.60]	0.481	1.10 [0.78, 1.56]	0.577	1.06 [0.75, 1.50]	0.747
Q3	1.28 [0.92, 1.79]	0.149	1.19 [0.85, 1.68]	0.304	1.09 [0.77, 1.54]	0.638
Q4	1.72 [1.26, 2.37]	0.001	1.57 [1.14, 2.17]	0.006	1.37 [0.99, 1.92]	0.063
*P* for trend	< 0.001		< 0.001		0.050	
**Heart problems**						
hs-CRP/HDL-C ratio per IQR	1.007(0.983,1.032)	0.560	1.006(0.982,1.031)	0.626	1.006(0.982,1.031)	0.628
Q1	ref		ref		ref	
Q2	0.98 [0.75, 1.29]	0.892	0.97 [0.73, 1.27]	0.806	0.96 [0.73, 1.27]	0.768
Q3	1.39 [1.08, 1.80]	0.012	1.37 [1.06, 1.78]	0.016	1.35 [1.04, 1.76]	0.026
Q4	1.33 [1.03, 1.72]	0.031	1.29 [1.00, 1.68]	0.053	1.28 [0.98, 1.67]	0.074
*P* for trend	< 0.001		< 0.001		0.013	

hs-CRP, high-density C-reactive protein; CVD, cardiovascular disease; HDL-C, high-density lipoprotein cholesterol; IQR, interquartile range; Q1, the 5th of the hs-CRP/HDL-C ratio; Q2, the 35th of the hs-CRP/HDL-C ratio; Q3, the 65th of the hs-CRP/HDL-C ratio; Q4, the 95th of the hs-CRP/HDL-C ratio

^a^ Model 1 evaluated the basic connection between the hs-CRP/HDL-C ratio and incident CVD.

^b^ Model 2 further controlled for sex, education level, residence location, and marital status

^c^ Model 3 further corrected for health behaviours (smoking status, alcohol intake and sleep duration) and physical and chemical indicators (LDL-C, blood glucose, uric acid, and blood pressure levels)

Likewise, research has revealed that the hs-CRP/HDL-C ratio is likely to contribute to the occurrence of stroke. According to Model 3, new strokes were associated with an increase in the hs-CRP/HDL-C ratio. Nevertheless, the differences in the outcomes were not significant (*P* for trend = 0.05).

Specifically, for heart problems, the correlation seemed to follow a “U” shaped trend of increasing and decreasing values. The results indicated that, compared with participants with an hs-CRP/HDL-C ratio in the 5th percentile (Q1), those with an hs-CRP/HDL-C ratio in the 65th percentile (Q3) were most susceptible to newly emerging cardiac issues (OR = 1.35, 95% CI 1.04–1.76; *p* = 0.026). Furthermore, after considering possible factors that may influence the results in Model 3 (*p* for trend = 0.013), the cumulative incidence rates of heart disease increased with increasing hs-CRP/HDL-C ratios.

**Fig. 2:**
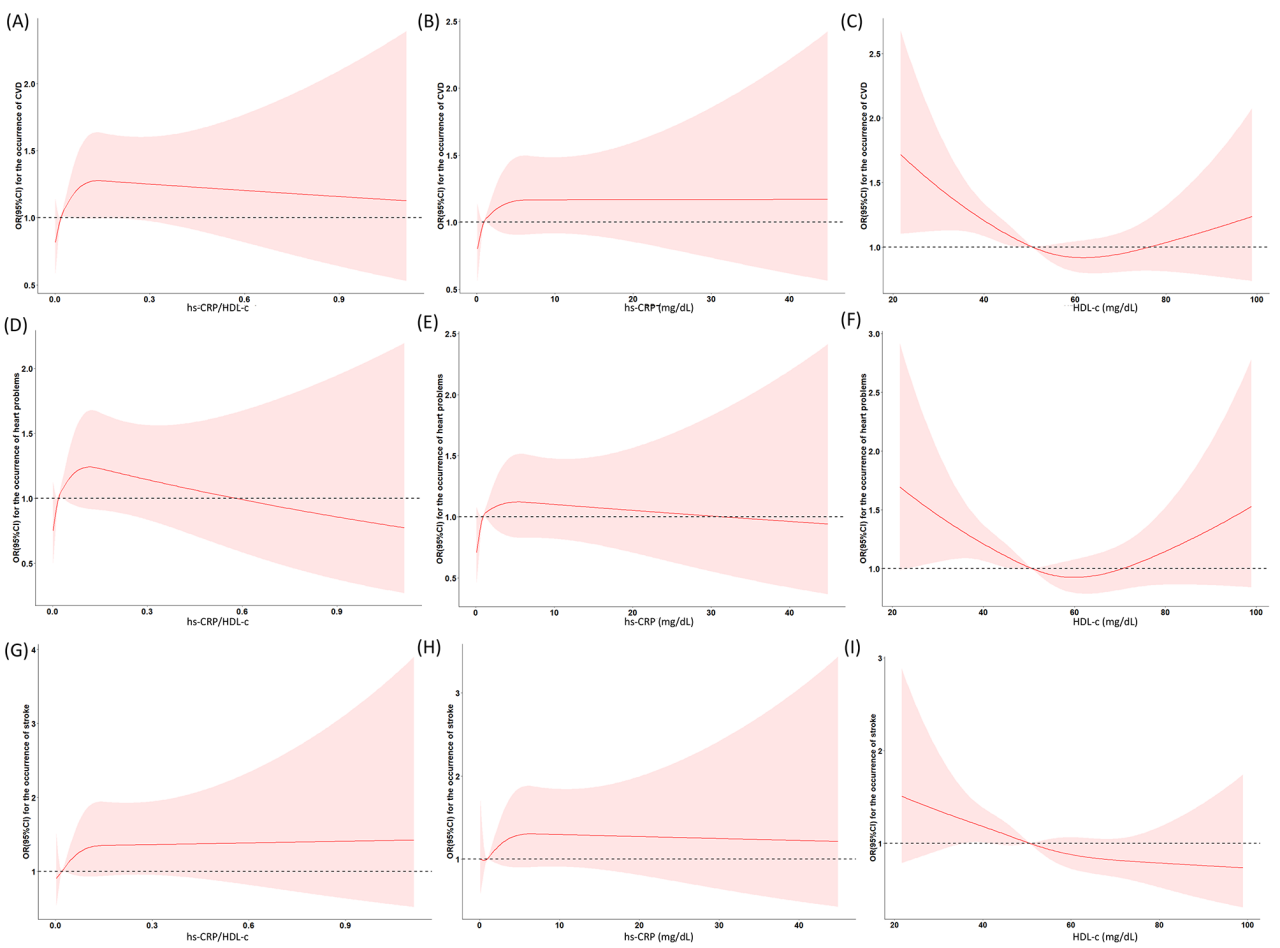
The cubic spline diagram of dose‒response associations: the dose‒response relationships between the hs-CRP/HDL-C ratio and the risk of newly developing CVD (**A**), heart issues (**D**) and stroke (**G**); the relationships between the hs-CRP concentration and the incidence of newly developed CVD (**B**), heart issues (**E**) and stroke (H); and the relationships between the HDL-C concentration and the incidence of newly developed CVD (**C**), heart issues (**F**) and stroke (**I**)

The dose‒response associations between the hs-CRP/HDL-C ratio and the incidence of CVD, stroke or heart issues are shown in Fig. 2 for all studies. According to the findings, there are nonlinear dose‒response associations between the hs-CRP/HDL-C ratio and newly developed CVD, stroke, and cardiac issues (*P*_overall_ < 0.05, _Pnonlinear_ < 0.05). Similarly, hs-CRP and HDL-C levels were significantly different (*P*_overall_ < 0.05, _Pnonlinear_ < 0.05).

### Stratified analysis

**Fig. 3:**
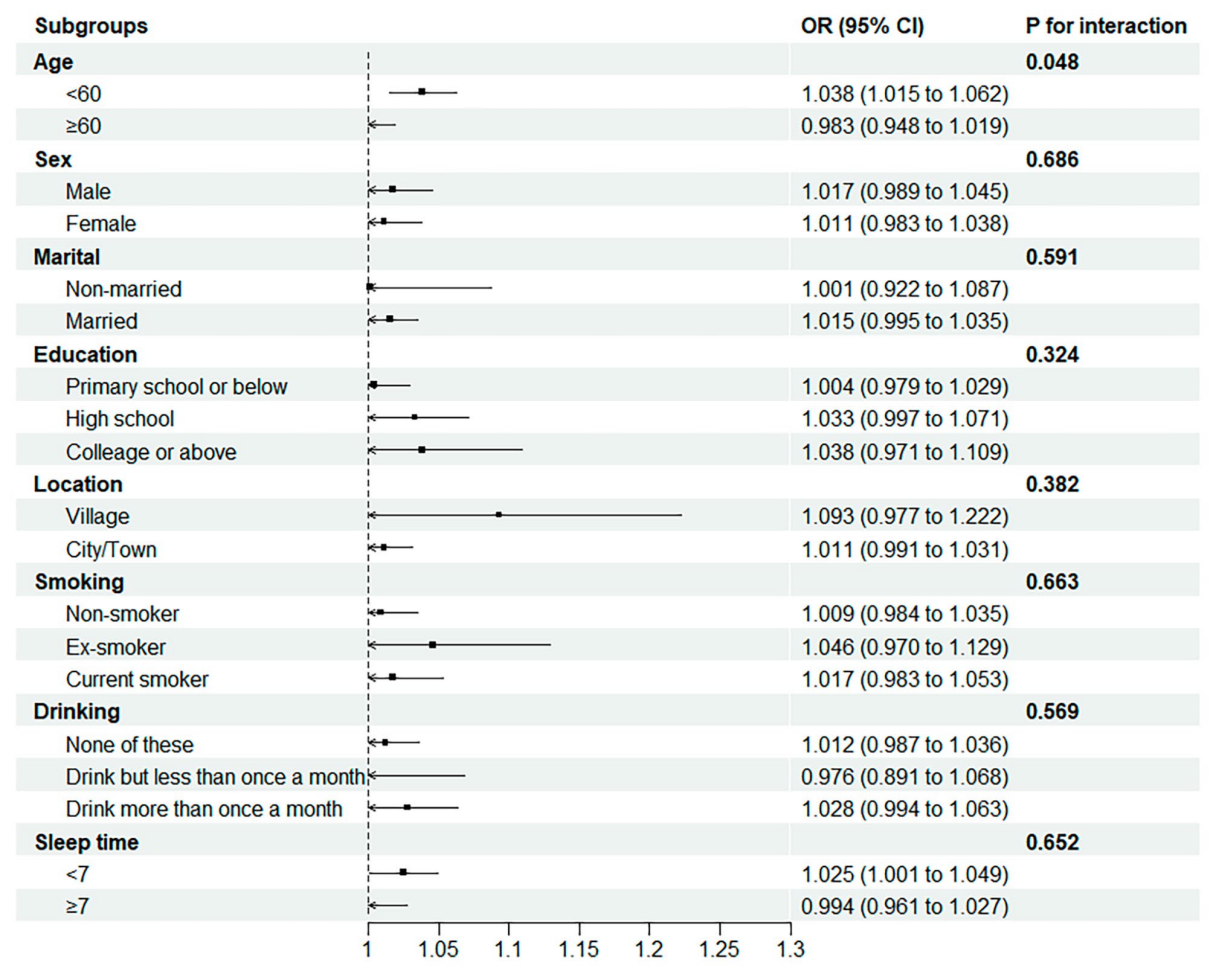
Subgroup analysis of the relationship between the hs-CRP/HDL-C ratio and the occurrence of CVD based on socioeconomic traits and medical history

Based on their medical histories and socioeconomic characteristics, participants were split into a number of subgroups to determine whether there were variations observed in the relationships between the hs-CRP/HDL-C ratio and the occurrence of CVD (see Fig. 3.). Age and the hs-CRP/HDL-C ratio were significantly related (*P* = 0.048). In particular, every IQR increase in the hs-CRP/HDL-C ratio was related to a 3.8% (95% CI 1.5%-6.2%) increased chance of CVD for participants under the age of 60 years, whereas there was no correlation for individuals aged 60 years or older.

### Incremental predictive value of the hs-CRP/HDL-C ratio

As shown in Table 3, adding the hs-CRP/HDL-C ratio to the basic model improved the C statistic estimate for incident CVD (0.630 vs. 0.628). However, the results were not statistically significant. Considering the limited impact of a single indicator on the C statistic estimate, the old and new models using the NRI and IDI were evaluated at the same time. A significant improvement in the NRI was found, in which CVD incidence increased by 0.0825 (*P* = 0.012) and the incidence of heart problems increased by 0.0938 (*P* = 0.020). The new model demonstrated enhanced predictive capabilities for NRI values above 0. Therefore, the significant increase in the continuous NRI suggested that incorporating the hs-CRP/HDL-C ratio into the basic model improved the accuracy of predicting both overall CVD incidence and heart problem incidence. Differences between IDI values in the old and new models were not statistically significant.

**Table 3:** Incremental predictive value of CRP/HDL-C

	C statistic Estimate (95% CI)	*P* value	NRI (continuous) Estimate (95% CI)	*P* value	IDI Estimate (95% CI)	*P* value
**CVD**						
Basic model	0.6276(0.6071,0.6481)		**ref**		**ref**	
Basic model + hs-CRP/HDL-C	0.6289(0.6085,0.6493)	0.3586	0.0825(0.0183,0.1468)	0.01181	0.0001(-0.0001,0.00002)	0.95889
**Stroke**						
Basic model	0.6901(0.6613,0.7189)		**ref**		**ref**	
Basic model + hs-CRP/HDL-C	0.6918(0.6633,0.7204)	0.232	0.0815(-0.0137,0.1767)	0.09344	-0.0002(-0.0006,0.0010)	0.6874
**Heart problems**						
Basic model	0.6157(0.5904,0.6409)		**ref**		**ref**	
Basic model + hs-CRP/HDL-C	0.6162(0.5910,0.6414)	0.4339	0.0938(0.015,0.1725)	0.01965	0.000(-0.0001,0.0001)	0.95889

CVD, cardiovascular disease; HDL-C, high-density lipoprotein cholesterol; hs-CRP, high-sensitivity C-reactive protein; IDI, integrated discrimination improvement; NRI, net reclassification improvement

## Discussion

This prospective cohort study included 6,554 eligible participants and examined baseline and follow-up data. A significant association was found between the likelihood of CVD and the ratio of hs-CRP to HDL-C levels.

The primary purpose of utilizing indices for inflammatory-lipid complexes in CVD prediction is to evaluate the extent of disease and vulnerability in individuals with CVD, assess the level of cardiovascular risk in patients without existing CVD, determine the likelihood of developing metabolic syndrome, and examine the impact of inflammation and dyslipidaemia on growth and development [26]. It is generally established that HDL-C can protect against CVD, which is recognized in medicine as a stand-alone risk factor for CVD [27–29]. Recent studies have revealed that the ratio of LDL-C to HDL-C levels has the potential to serve as a predictive indicator for both disease risk and prognosis [30–32]. In addition, researchers have shown that high TC/HDL-C and TG/HDL-C ratios can be considered risk factors for CHD [33]. Furthermore, there have been claims stating that non-HDL-C is a more effective indicator in predicting the likelihood of CHD, and relevant guidelines suggest treating it accordingly [33, 34].

Previous studies have shown that the inflammatory response promotes the progression of atherosclerosis [35–37], and the CANTOS study further confirmed the use of the monoclonal antibody canakinumab for anti-inflammatory intervention. By targeting interleukin (IL)-1β within the CRP/IL-6/IL-1 axis, this intervention has shown promising results in improving patient prognosis [38]. CRP, an easily accessible serum inflammatory marker, has been demonstrated to be connected to the long-term prognosis of AMI and CAD patients in previous research [39, 40]. According to research by Schakelaar MY et al., an automated chemical analyser can be used to measure CRP levels effectively from dried blood spots obtained through finger sticks, showing efficacy in identifying individuals with an elevated susceptibility to CVD [41]. Research has also revealed that hs-CRP is a biomarker of inflammation and is linked to a greater likelihood of developing atherosclerotic CVD [42, 43]. The combination of hs-CRP levels and lipid markers, such as the hs-CRP and triglyceride-glucose [44] and the hs-CRP and remnant lipoprotein cholesterol indices [45], has also been used clinically as a prognostic predictor of CAD.

According to the present study, there is a correlation between the hs-CRP/HDL-C ratio and the likelihood of developing CVD. The interplay between lipid metabolism and inflammation is of utmost importance in the development of CVD. Reverse cholesterol transport, antioxidant, anti-inflammatory, endothelial/vasodilatory, antithrombotic, and cytoprotective actions are the primary biological attributes of HDL-C [46]. In particular, the anti-inflammatory properties of HDL prevent its oxidative modification of LDL [32]. By reducing the expression of adhesion molecules on endothelial cell surfaces, HDL has the ability to counter inflammation. The proteins that are impacted include P-selectin, E-selectin, intercellular adhesion molecule-1 (ICAM-1), and vascular cell adhesion molecule-1 (VCAM-1). HDL prevents T lymphocytes and monocytes from adhering to the vascular endothelium and migrating to atherosclerotic areas [47, 48]. Conversely, inflammation leads to a decrease in and structural changes in HDL. Additionally, HDL-related proteins, enzymes, and transfer proteins that are involved in HDL metabolism and function undergo significant alterations [49]. However, further investigation is necessary to validate its accuracy and exact methodology.

### Study strengths and limitations

In previous studies, risk prediction models for CVD have focused mostly on independent lipid indices or independent inflammatory indices [7–9, 14–18]. This study considered the correlation between lipid metabolism and inflammatory metabolism and focused on inflammation-lipid composite indices in peripheral blood from CHD and AMI patients, thereby improving the accuracy of clinical prognosis prediction. Hs-CRP and HDL-C levels are easy to obtain, feasible and inexpensive are highly important for clinicians, especially primary care doctors, in predicting the incidence of CVD in the population.

Nevertheless, there are certain limitations associated with this study. First, this study had a long period of data collection, and medication data were not collected from the patients, which may have interfered with the results. This study did not exclude patients who had hypertension at baseline, and patients’ use of oral antihypertensive medications may affect the accuracy of the hs-CRP and HDL-C levels. Second, this study was limited to middle-aged and elderly Chinese patients. The study performed validation in a wider population is needed in the future. Third, the diagnosis of stroke depended on the subjects’ self-reports, which might cause bias. Finally, the CHARLS was carried out early and has not addressed new lipid indicators of interest in recent years, such as Lp(a), which is a well-known factor contributing to the risk of CVD [50].

## Conclusion

According to this cohort study, a high hs-CRP/HDL-C ratio is a significant risk factor for CVD, new stroke, and heart problems. Early intervention in patients with increased hs-CRP/HDL-C ratios may further reduce the incidence of CVD, in addition to focusing on independent lipid markers or independent inflammatory markers. Additional investigations are needed to gain a comprehensive understanding of the fundamental underlying mechanisms involved.

### Electronic supplementary material

Below is the link to the electronic supplementary material.

Supplementary Material 1

## Data Availability

The CHARLS website provides access to the data that support the findings of this study (http://charls.pku.edu.cn/en).

## Abbreviations

AMI: Acute myocardial infarction
ANOVA: Analysis of variance
CAD: Coronary artery disease
CHARLS: The China Health and Retirement Longitudinal Study
CI: Confidence interval
CRP: C-reactive protein
CVD: Cardiovascular disease
HDL-C: High-density lipoprotein cholesterol
hs-CRP: High-sensitivity C-reactive protein
ICAM-1: Intercellular adhesion molecule-1
IDI: Integrated discrimination improvement
IQR: Interquartile rang
K-W test: Kruskal‒Wallis test
LDL-C: Low-density lipoprotein cholesterol
NRI: Net Reclassification Improvement
OR: Odds ratio
Q: Quartile
TG: Triglyceride
VCAM-1: Vascular cell adhesion molecule-1
